# A Priori Dietary Patterns, Physical Activity Level, and Body Composition in Postmenopausal Women: A Cross-Sectional Study

**DOI:** 10.3390/ijerph19116747

**Published:** 2022-05-31

**Authors:** Benedetta Bendinelli, Elisa Pastore, Miriam Fontana, Ilaria Ermini, Melania Assedi, Luigi Facchini, Andrea Querci, Saverio Caini, Giovanna Masala

**Affiliations:** 1 Clinical Epidemiology Unit, Institute for Cancer Research, Prevention and Clinical Network (ISPRO), Via Cosimo il Vecchio 2, 50139 Florence, Italy; b.bendinelli@ispro.toscana.it (B.B.); e.pastore@ispro.toscana.it (E.P.); m.fontana@ispro.toscana.it (M.F.); a.querci@ispro.toscana.it (A.Q.); g.masala@ispro.toscana.it (G.M.); 2 Cancer Risk Factors and Lifestyle Epidemiology Unit, Institute for Cancer Research, Prevention and Clinical Network (ISPRO), Via Cosimo il Vecchio 2, 50139 Florence, Italy; i.ermini@ispro.toscana.it (I.E.); m.assedi@ispro.toscana.it (M.A.); l.facchini@ispro.toscana.it (L.F.)

**Keywords:** postmenopausal women, dietary pattern, physical activity, body composition

## Abstract

Midlife weight gain and fat distribution changes increase the risk of age-related pathologies. We aimed to explore, in a series of 388 healthy postmenopausal women living in Tuscany, Central Italy, the relationship between three *a priori* dietary patterns, the level of physical activity (PA), and four body composition measures: body mass index (BMI), percent fat mass (%FM), percent muscle mass (%MM), and waist circumference (WC). Detailed information on lifestyle, including the amount of recreational and household PA, sitting time, and dietary habits were collected through detailed questionnaires, and adherence scores to Greek Modified Mediterranean Diet, Italian Mediterranean Diet (IMD), and Dietary Approaches to Stop Hypertension diet were calculated. The %FM and %MM were estimated via TANITA MC-780MA analyzer. WC and BMI were measured according to standard international protocols. Cross-sectional adjusted regression models showed that increasing adherence to IMD was inversely associated with BMI, %FM, and WC, and directly associated with %MM. Higher levels of recreational PA were associated with lower %FM, BMI, and WC and with higher %MM values. Higher levels of sitting time were associated with higher %FM, BMI, and WC, and lower %MM. Dietary habits and moderate PA confirm their central role in maintaining good health even in menopausal women.

## 1. Introduction

Worldwide, after age 45, obesity rates are higher for women than for men [1]. Postmenopausal weight gain has been related to a combination of non-modifiable and modifiable factors such as ageing, decreased estrogen levels after menopause, altered metabolism, excessive caloric consumption, and sedentary lifestyle [2,3]. Along with ageing, women’s body composition undergoes changes: lean mass decreases, resulting in decreased basal metabolic rate and energy expenditure [4,5], and fat mass increases, even in women who do not experience an increase in total body weight, with a shift of fat toward a visceral distribution [5,6]. Metabolic and fat distribution changes associated with midlife weight gain increase the risk of cardiovascular disease and type II diabetes, hypertension, and metabolic syndrome [3,7,8]. Therefore, the evaluation of the effect of modifiable factors on body composition parameters might be relevant for the prevention of these chronic diseases.

The etiology of obesity in middle-aged women is still not fully elucidated, although evidence clearly indicates that diet and physical exercise represent two of the modifiable factors that influence weight and some aspects of body composition [7,9,10,11]. As reported in previous literature, physical activity and diet may play an important role in the prevention of obesity in postmenopausal women, probably in a synergistic manner. In particular, in a study by JA Martinez, it was found that postmenopausal women who met the Recommended Daily Allowance (RDA) of protein associated with a good level of PA had better results in body composition [12].

Regarding diet, in recent years, the focus has shifted from individual foods and nutrients to dietary patterns, as they can provide a snapshot of an individual’s eating habits.

Moreover, the role of physical exercise and of sedentary habits should be also evaluated, especially since, generally, physical activity decreases with aging.

We combined these two points and attempted to assess how dietary habits as a whole and physical activity affect body composition (as measured by bioimpedance test) in women at a stage of life when all these parameters change for various reasons (less inclination to cook, less agility, biological factors).

Therefore, the aim of the present cross-sectional study was to explore, in a series of healthy postmenopausal women living in Tuscany, Central Italy, previously enrolled in the European Prospective Investigation into Cancer and Nutrition (EPIC) Florence cohort, the relationship between adherence to three a priori dietary patterns, the level of physical activity (PA), and sedentary time, and each of the following four body composition measures: body mass index (BMI), percent fat mass (%FM), percent muscle mass (%MM), and waist circumference (WC).

## 2. Materials and Methods

### 2.1. Study Population

The study was carried out in postmenopausal women previously enrolled in the Florence section of the EPIC study.

The methodology of the EPIC study has been published elsewhere [13]. Briefly, in 1993–1998 detailed information on reproductive history, smoking, and alcohol drinking history, educational level, physical activity habits, and medical history were collected for about 10,000 women through a standardized lifestyle questionnaire (LSQ). Body weight, body height, and waist and hip circumferences were measured by trained nurses according to an international standard protocol. Data on frequency of consumption of 188 foods and drinks and usual portion size were obtained through a validated self-administered Food Frequency Questionnaire (FFQ) specifically developed to capture Italian dietary habits [13].

In 2019, a representative sample of the Florence EPIC cohort of 403 women (out of a total of 631 women invited by an informative letter followed by telephone contacts) participated, as part of a specific project called FEDRA (Florence-EPIC Digital Mammographic breast density and breast cancer risk assessment), in a follow-up outpatient visit; the purpose was to update information on lifestyle and diet (through the same FFQ and LSQ used in the previous EPIC enrollment) and anthropometric measurements (body weight, waist and hip circumferences) and to collect body composition measurements.

The visit was performed by trained nurses according to an international standard protocol. The follow-up was approved by the local Ethics Committee. All procedures performed were in accordance with the ethical standards of the institutional and national research committee and with the 1964 Helsinki declaration and its later amendments or comparable ethical standards. At the beginning of the visit, all study participants signed an informed consent and gave permission to use the data collected during the study.

### 2.2. Dietary Assessment

Updated dietary information was collected by the EPIC FFQ administered by trained nurses. It collects information on dietary habits over the past year and includes 299 questions in 16 sections: pasta/rice, soup, meat (excluding salami and other cured meats), fish, raw vegetables, cooked vegetables, eggs, sandwiches, salami and other cured meats, cheese, fruit, bread/wine, milk/coffee/cakes, herbs/spices, cooking methods, other general information relating to changes to the habitual diet in the last year, meals consumed at home and outside home, places of origin of the fresh vegetables, and possession of a freezer. The number of times a given food item was consumed (per day, week, month, or year) was also asked. The quantity of the food consumed was assessed from the respondent’s selection of an image of a food portion, or using a predefined standard portion (e.g., glass or cup) when no image was available. For sauces, meat, fish, and vegetables, there were questions on cooking method and type of fat used for preparation and cooking. For tomatoes and other specific foods, whose intake is strongly dependent on season, intake was assessed separately in and out of the main cropping season [14]. Data from FFQ were converted into frequencies of consumption and average daily quantities of foods, energy, and nutrients consumed, by linkage to specific Italian Food Tables [15].

### 2.3. Physical Activity and Sitting Time Assessment

Updated PA habits of participants were collected through the EPIC LSQ (administered by trained nurses), which includes a specific section aimed to assess PA habits in the last year both in occupational and leisure time settings [16,17]. Women were asked if they currently had a paid full-time or part-time job and to classify their current occupation as sedentary, standing, manual, or heavy manual. They were also requested to indicate the number of hours they spent during the week, separately for summer and winter periods, in leisure time activities, in particular walking (including walking to the work place, shopping, and walking for pleasure), cycling (including cycling to the work place and during leisure time), gardening, and fitness (including gym activities, swimming, playing tennis, running, etc.). Subjects were requested also to indicate, independently of the season, the weekly hours of do-it-yourself activities, the daily number of hours spent in housework, and finally the number of floors of stairs climbed per day. The hours spent in each activity were multiplied by the respective metabolic equivalent (MET) values according to the “Compendium of Physical Activities” from Ainsworth BE et al., in which a comprehensive list of MET intensities of all occupational, household, and recreational activities is reported [18]. The MET-hour products were summed to give a weekly MET score of recreational activities (walking, cycling, and fitness) and a weekly MET score of household activities (do-it-yourself, housework, gardening, and stair climbing). All MET-hour products were summed to give a total weekly MET score, which represented the amount of energy expended during an average week for leisure activities. Sitting time takes into account the amount of free time (i.e., outside of work) spent sitting during the day in activities such as sewing, reading, watching television, using computers, etc.; the variable is divided into the following classes: ≤2 h/day; from >2 to ≤4 h/day; >4 h/day.

### 2.4. Lifestyle and Diseases Data Collection

The EPIC LSQ was also used to update information on smoking status at follow-up (current, former, or never-smokers), and history of diseases and drug use occurred after the EPIC enrollment, including the diagnoses of cancer, diabetes, myocardial infarction, stroke, hypertension, and high cholesterolemia. Information on menopausal status and use of hormonal replacement therapy (HRT) was also updated.

### 2.5. Anthropometric and Body Compositions Measures

At follow-up, participants’ weight (kg), fat mass (FM, kg), percentage of total body body fat (%FM), muscle mass (MM, kg), and percentage of total body muscle mass (%MM) were estimated via the bioelectrical impedance body composition analyzer TANITA MC-780MA (Tanita Corp., Tokyo, Japan) [19]. The latter consists of a stand-alone unit where age, gender, and height of participants are inputted by the operator. During the impedance measurement, participants wear light clothing and must step onto a special platform with bare feet and take the handles with both hands alongside the body. Body mass index (BMI) was calculated as weight (kg)/height^2^ (m). Waist and hip circumferences (cm) measures were collected by trained operators according to a standard international protocol.

### 2.6. A Priori-Defined Diet Scoring Protocols

Mediterranean Diet Score (MDS): a score indicating the degree of adherence to the Greek Modified Mediterranean Diet GMMD proposed by Trichopoulou et al. [20]. A value of 0 or 1 was assigned to each of the nine score components according to the median cut-off for daily consumption. For the following components: vegetables, legumes, fruits and nuts, cereals, fish, and ratio of mono-/polyunsaturated fatty acids to saturated fatty acids, consumption above the relative study medians received 1 point. For dairy products, meat, and meat product components, consumption below the median received 1 point. As regards alcohol intake, women who consumed 5–25 g/day received 1 point. The total MDS ranged from 0 to 9 points.

Italian Mediterranean Index (IMI): a scale indicating the degree of adherence to the Italian Mediterranean Diet (IMD), a dietary pattern developed to take into account Italian eating habits [21]. It includes six typically Mediterranean foods (pasta, vegetables, fruit, legumes, olive oil, and fish) and five food components not typically present in the Mediterranean diet (soft drinks, alcohol, butter, red meat, and potatoes). A value of 0 or 1 was assigned to each of the 11 components. The attribution of individual scores is done according to sex-specific tertiles of daily consumption for typically Mediterranean foods, and 1 point was assigned to participants in the third tertile of consumption. For extra Mediterranean foods, 1 point was assigned to participants in the first tertile of consumption. The total IMI ranged from 0 to 11 points.

Dietary Approaches to Stop Hypertension (DASH) score: To assess the degree of adherence to the DASH diet [22] we applied the protocol designed by Fung T et al. [23], which includes a set of eight foods: fruits, vegetables, nuts and legumes, low-fat dairy products, whole grains, sweetened beverages, sodium, and red/processed meat. A component score ranging from 1 to 5 points was assigned to fruits, vegetables, nuts and legumes, low-fat dairy products, and whole grains, according to the specific quintile of daily consumption. For sodium, red/processed meats, and sweetened beverages, the component score ranging from 1 to 5 points was assigned in decreasing order considering the quintile of consumption. The scores assigned to each food were then summed up to obtain an overall DASH score ranging between 8 and 40 points.

### 2.7. Statistical Analyses

According to follow-up measurements, subjects have been divided in three BMI classes: underweight/normal (BMI < 25), overweight (25 ≤ BMI < 30), obese (BMI ≥ 30). Three categories of educational levels (none/primary/secondary school, professional/high school, master/doctor) and three smoking status classes (current smokers, ex-smokers, or never-smokers at follow-up) were also created.

Means and standard deviation (SD) and distributions (N, %) of study population according to principal anthropometric and lifestyle characteristics were calculated for continuous and categorical variables, respectively.

For each of the a priori dietary patterns and according to follow-up dietary information, subjects were categorized into tertiles of the total score, to be interpreted as “low-adherence”, “medium adherence”, and “good adherence”, as reported in a previous paper [21].

Subjects were also categorized in tertiles of recreational and household physical activity. For sitting time, the following categories were used: 0–2 h/day; 3–4 h/day; >4 h/day.

To evaluate the association between adherence to the three a priori dietary patterns and %FM, %MM, BMI, and WC, we fitted cross-sectional regression models adjusted for age at follow-up (years, continuous), body height at follow-up (cm, continuous), smoking habits (dummy variables for current and ex-smokers at follow-up), parity (any/no children at follow-up), educational level (dummy variables for professional/high school, master/doctor) and number of years from menopause to follow-up (years, continuous). The total scores of the three *a priori* dietary patterns were analyzed as dummy variables of the adherence categories (“low adherence” as reference category). The regression coefficients (β) represent the adjusted difference in %FM, %MM, BMI (kg/m^2^), and WC (cm) between the relative adherence category and the reference category. Linearity of trends across adherence categories was tested by treating categories as a continuous variable. To make it possible to compare results obtained with the different patterns, the regression models were also applied to the continuous standardized values of the total scores of the dietary patterns. The standardized values were obtained by subtracting the mean of each pattern from the value of each subject and then dividing by standard deviation. The regression coefficients (β) represent the adjusted difference in %FM, %MM, BMI (kg/m^2^), and WC (cm), between two consecutive standardized values.

Regression model further adjusted for sitting time (categorical variable for ≤2 h/day; >2–≤4 h/day; >4 h/day), recreational activity (MET-hours/week, continuous) and household activity (MET-hours/week, continuous) were also performed.

To evaluate the association between leisure time PA and %FM, %MM, BMI, and WC (cm), regression models were performed, adjusted for age at follow-up, body height at FU, smoking habits, children, educational level, number of years from menopause to follow-up, and total IMI score at follow-up (continuous). Tertiles of recreational and household activities and categories of sitting time were analyzed as dummy variables with the lowest category as reference. The regression coefficients (β) represent the adjusted difference in %FM, %MM, BMI (kg/m^2^), and WC (cm) between the relative PA category and the reference category. Linearity of trends across PA categories was tested by treating categories as a continuous variable. The regression models were also applied to the continuous standardized values. Regression models further adjusted for total IMD score were also performed.

Additional models adjusted for presence of hypertension, high cholesterolemia, diabetes, cardiovascular diseases, and cancer were also run.

## 3. Results

The full set of anthropometric and body composition measurements and the updated EPIC LSQ information was available for 388 (96.3%) out of 403 postmenopausal women attending the follow-up visit. Updated dietary information from EPIC FFQ was available for 362 women.

Distribution at follow-up of the main characteristics of the 388 EPIC-Florence participants are reported in Table 1.

All participants were postmenopausal with a mean age of 70.3 (SD 6.1) years. Mean age at menopause was 50.5 (SD 4.1) years, with a mean of 19.8 (SD 7.7) years between menopause and follow-up. Ninety-three percent of the women were or had been married at follow-up, 82.7% had at least one child, and 90.3% were ex- or never-smokers. Among the participants, 41.7% were hypertensive, 58.8% reported high cholesterolemia, and 6.4% reported a diagnosis of diabetes. Mean %FM was 33.1 (SD 6.6), mean %MM was 63.5 (SD 6.2), mean WC was 85.0 (SD 11.7), and mean BMI was 26.8 (SD 4.8), with 59.3% of women classified as overweight or obese.

### 3.1. Dietary Patterns

The associations between the adherence to the three *a priori* dietary patterns and body composition are shown in Table 2.

In models not adjusted for PA variables, “good adherence” as compared to “low adherence” was associated with a significantly lower BMI when IMD (β −2.19, 95%CI −3.53; −0.85, *p* trend 0.002) and GMMD (β −1.66, 95%CI −2.91; −0.41, *p* trend 0.01) were considered, respectively. “Good adherence” as compared to “low adherence” was associated with significantly lower %FM for both IMD (β −3.62, 95%CI −5.48; −1.76, *p* trend < 0.0001) and GMMD (β −1.89, 95%CI −3.63; −0.14, *p* trend 0.04). “Good adherence” as compared to “low adherence” was associated with significantly higher %MM for both IMD (β 3.42, 95%CI 1.66; 5.19, *p* trend < 0.0001) and GMMD (β 1.77, 95%CI 0.12; 3.43, *p* trend 0.04). “Good adherence” as compared to “low adherence” was associated with significantly lower WC for IMD (β −5.31, 95%CI −8.60;−2.02, *p* trend 0.003), GMMD (β −3.48, 95%CI −6.56; −0.41, *p* trend 0.03), and DASH (β −3.53, 95%CI −6.51; −0.55, *p* trend 0.02).

Analyses based on continuous standardized score adherence showed an inverse association of IMD and GMMD with %FM (β −1.25 and −0.70, respectively, all *p* values < 0.05), with BMI (β −0.88 and −0.59, respectively, all *p* values < 0.05) and a direct association with %MM (β 1.18 and 0.66, respectively, all *p* values < 0.05). Regarding WC, analyses based on continuous standardized score adherence showed an inverse association with IMD (β −2.13, *p* value < 0.05) and with DASH (β −1.35, *p* value < 0.05). In models adjusted for PA, the reported associations of IMD with %FM, %MM, BMI, and WC persisted. No significant association with BMI, %FM, or %MM emerged for adherence to DASH.

### 3.2. Leisure Time PA and Sitting Time

The associations between leisure time PA and body composition characteristics are reported in Table 3.

In models also adjusted for IMD total score, recreational PA was significantly associated with lower BMI and lower WC (β −2.11 and −4.24 respectively for “third tertile” vs. “first tertile”, all *p* values < 0.03, all *p* for trend < 0.05). Sitting time was significantly associated with higher BMI, higher %FM, higher WC, and lower %MM (β 2.30, 2.77, 5.41 and −2.62, respectively for “third category” vs. “first category”, all *p* values < 0.05, all *p* for trend ≤ 0.005).

Analyses for continuous standardized MET-hours/week of recreational PA showed an inverse association with %FM (β −0.89, 95%CI −1.62; −0.15), an inverse association with BMI (β −0.91, 95%CI −1.43; −0.39), an inverse association with WC (β −1.86, 95%CI −3.15; −0.58), and a direct association with %MM (β 0.84, 95%CI 0.15; 1.54).

Analyses for classes of sitting time showed a direct association with %FM (β 0.99, 95%CI 0.18; 1.81), a direct association with BMI (β 0.79, 95%CI 0.21; 1.37), a direct association with WC (β 1.72, 95%CI 0.30; 3.15), and an inverse association with %MM (β −0.94, 95%CI −1.71; −0.17).

Household PA was not significantly associated with BMI, %FM, %MM, or WC.

Additional models adjusted for presence of hypertension, high cholesterolemia, diabetes, cardiovascular diseases, and cancer showed similar results.

## 4. Discussion

In this cross-sectional study on postmenopausal women, a significant association between the adherence to healthy dietary patterns, the level of leisure-time physical activity, and improved body composition parameters emerged.

In this paper we investigated two a priori dietary patterns specifically developed to capture the dietary habits of Mediterranean populations, the Greek Modified Mediterranean Diet (GMMD) and the Italian Mediterranean Diet (IMD) [20,21], as well as the Dietary Approaches to Stop Hypertension (DASH), which is used worldwide as a standard for good-quality diets [22].

We found that “good adherence” to IMD and GMMD compared with “low adherence” was associated with significantly lower BMI, %FM, and WC and with significantly higher MM%. “Good adherence” as compared to “low adherence” was associated with lower WC for DASH, as well. In models also adjusted for PA variables, the associations with adherence to IMD persisted for all outcomes. Adjusted analyses for adherence score in continuous showed that increasing adherence scores to IMD were inversely associated with BMI, %FM, and WC and directly associated with %MM.

We also investigated the association between PA and body composition and found results consistent with those of several studies available in the literature: the upper tertile of recreational PA such as walking, bicycling, and swimming (with a range of 28.5 to 171.0 MET-hours/week), as compared to the lower tertile, was associated with lower %FM, BMI, and WC and with higher %MM values; higher levels of sitting time were associated with higher %FM, higher BMI values, higher WC values, and lower %MM values.

Regarding PA, our results on sitting time are consistent with those reported in the literature. A study published in 2019 [24] reported an improvement in indices of obesity and metabolic syndrome in adults who had replaced 30 min/day in bed with 30 min/day of light or moderate PA; these results are in agreement with our results on sitting time, which was found to be directly associated with body composition indices of obesity. Sitting time is an interesting variable to evaluate in our population of old women (average age 70.3 years), a stage of life characterized by modification of physical activity patterns and a reduction of daily activities.

A study by Kyle UG et al. conducted on 3853 individuals aged 15–64 years investigated the association between different levels of PA and body composition. It was seen that “physically active” people (>3 h/week of PA such as jogging, swimming, tennis, and gymnastics) had a higher percentage of lean mass than sedentary people (<3 h/week of PA), while sedentary people had higher %FM values. In addition, despite ageing, the increase in %FM was slower in “physically active” people [25].

A study conducted on men and women with an average age of 65 years showed, in participants who increased moderate-to-vigorous PA by 1 h/day, a significant inverse association with BMI, waist circumference, and fat mass and a significant direct association with bone mass and lower-limb muscle strength. Participants who increased sedentary time, on the other hand, had higher fat mass and waist circumference. In this study, light physical activity was not significantly associated with any outcome [26]. Results on BMI, WC, and fat mass agree with ours; however, in the higher tertiles of recreational physical activity, we also found a direct association with %MM.

It is well-known by now that physical activity plays a key role in maintaining good body composition and health. What is interesting to note, however, is that in our study, even a moderate physical activity done during leisure time can have beneficial effects. These are encouraging findings especially in a population in which ageing can make it more difficult to perform intense physical activity.

About dietary patterns, it is interesting to note that despite the differences in the dietary components and in the scoring systems, the results obtained in our study are consistent and almost similar for the two Mediterranean dietary patterns, while the DASH is not associated with body composition parameters (except for a mild inverse association with waist circumference). The most relevant results on body composition, however, emerged from analyses with the IMD that specifically takes into account Italian dietary habits. Moreover, this food pattern is the only one to present “contrasting” food pairs, i.e., healthy vs. unhealthy foods (pasta-potatoes, olive oil-butter, fish-red meat), so that increased consumption of healthy foods is presumably correlated with reduced consumption of unhealthy foods. It should also be noted that the IMD score, by using the highest tertile of consumption for the allocation of points, allows a more efficient classification of subjects according to their food consumption. [21].

Notably, in the frame of the Fitness League Against MENopause COst (FLAMENCO) project, a cross-sectional analysis on 176 perimenopausal women aged 45–60 years and residing in southern Spain showed that a high adherence to the Mediterranean diet (calculated through the Mediterranean diet score proposed by Panagiotakos et al. [27]) was associated with a high ratio of hip to total fat mass (ratio of gynoid to total FM) and a low ratio of abdominal to hip fat mass (ratio of abdominal to gynoid FM). Moreover, in this study, an inverse association emerged between whole grains, nuts, dairy products, legumes, and extra virgin olive oil intake and percent fat mass, waist circumference, and BMI. Although these results were not strictly related to food patterns, almost all of the considered foods are also included in the patterns we used.

Similar results on the Mediterranean diet score proposed by Panagiotakos were obtained in a group of 481 postmenopausal women in a cross-sectional study in which higher levels of adherence to the dietary pattern under study were associated with lower levels of BMI, lower levels of waist circumference, and a lower waist-to-hip ratio [28]. Another recent study by Cespedes F. et al. [29], conducted on 67175 postmenopausal women during 3 years of follow-up, suggests that adherence to a healthy dietary pattern is an optimal strategy for containing increased total and abdominal adiposity. Specifically, they reported the association between improved diet quality, as assessed by greater adherence to four specific dietary patterns (Healthy Eating Index 2010, Alternate Healthy Eating Index 2010, Alternate Mediterranean Diet, and DASH), and reduced increases in waist circumference and abdominal fat (largely explained by reduced body weight gain), even over a 3-year period.

This study has several strengths: the accurate collection of dietary data obtained through a validated FFQ specifically developed for Italian dietary habits [14,30], the direct measurement of anthropometric indices by specialized personnel following specific standardized protocols, and the possibility of adjusting models for several lifestyle variables obtained from a validated LSQ. The analysis of body composition was performed using the TANITA MC-780MA analyzer, which is considered an adequate method to assess body composition in epidemiological studies among young and middle-aged women [19,31,32].

A first limitation of the study is the cross-sectional nature that precludes the attribution of causality to the results obtained. However, we can speculate that lifestyle habits do not rapidly change in this age range. We also performed analyses adjusted for diseases reported by participants that could have affected both lifestyle habits and body composition. The results did not materially change, thus reducing the possibility of a reverse causation bias. A second limitation concerns the PA assessment method. PA was reported by participants in a section of the LSQ and was not objectively measured as in other studies. However, we were able to rely on highly detailed, validated questionnaires [16]. In our analysis, we did not use visceral fat measurement obtained by TANITA as an outcome of body composition. This decision was driven by the fact that the visceral fat mass, differently by %FM and %MM, was not measured but estimated by the TANITA instrument with an indirect algorithm. The increase in visceral fat attributable to menopause is, however, a very interesting topic on which studies are not yet clear and concordant. For example, a study by Cespedes F. et al. found that adherence to the Mediterranean dietary pattern was correlated with lower values of visceral fat [29], while a study published in 2018 conducted on overweight women and men found no association between adherence to the Mediterranean diet and visceral fat [24]. Regarding PA, a study published by Kanaley JA reported that different levels of PA were associated with visceral fat in pre- and postmenopausal women [7]. In our study, waist circumference was used as an estimate of abdominal fat, and the results obtained were consistent with those found in the literature.

## 5. Conclusions

The identification and development of strategies to prevent or decrease body fat gain is of great importance. In our study, the focus on nutrition was on *a priori* dietary patterns rather than on individual foods or nutrients because the patterns are able to provide a complete picture of dietary habits and allow us to evaluate the combined effect of foods and nutrients. The high adherence to healthy dietary patterns, such as the Mediterranean Diet, contributes to determining a better body composition in postmenopausal women. Our results on recreational physical activity confirm that even in postmenopause, it is important to recommend at least a moderate level of physical activity. In this study, we confirm that dietary habits and physical activity play a central role in maintaining good health even in menopausal women. A healthy diet and an at least moderate amount of physical activity should therefore be particularly promoted in postmenopausal women to recover or maintain a healthy body composition and consequently to reduce the risk of age-related diseases.

## Figures and Tables

**Table 1:** Means (SD) and distribution (N, %) at follow-up of the main characteristics of the 388 EPIC-Florence participants with follow-up information.

	Mean (SD)/N (%) ^1^
**Demographic and lifestyle characteristics**	
Age at follow-up (years), mean (SD)	70.3 (6.1)
Age at menopause (years), mean (SD)	50.5 (4.1)
Time from menopause to follow-up (years), mean (SD)	19.8 (7.7)
One or more sons/daughters, N (%)	321 (82.7)
Marital status, N (%)	
Unmarried	26 (6.7)
Married/cohabiting	247 (63.7)
Divorced/separated	41 (10.6)
Widowed	73 (18.8)
Educational level, N (%)	
None/Primary/Secondary school	122 (31.4)
Professional/High school	175 (45.1)
Master/Doctor degree	85 (21.9)
Smoking status, N (%)	
Never-smoker	195 (50.3)
Former smoker	155 (40.0)
Current smoker	37 (9.5)
**Physical activity characteristics**	
Current job, N (%)	
Yes	56 (14.4)
Sedentary occupation	38 (9.8)
Standing occupation	14 (3.6)
Manual work	3 (0.8)
No	332 (85.6)
Housewife	44 (11.3)
Retired	281 (72.4)
Recreational physical activity (MET-hour/week), mean (SD)	25.5 (21.2)
Household physical activity (MET-hour/week), mean (SD)	50.1 (31.4)
Sitting time, N (%)	
≤2 h/day	170 (43.9)
>2; ≤4 h/day	162 (41.9)
>4 h/day	55 (14.2)
**Medical history**	
Hypertension, N (%)	162 (41.7)
Myocardial infarction, N (%)	5 (0.3)
Stroke, N (%)	3 (0.8)
High cholesterolemia, N (%)	228 (58.8)
Cancer (all sites), N (%)	9 (2.3)
Diabetes, N (%)	25 (6.4)
HRT, N (%)	7 (1.8)
**Anthropometric and body composition measures**	
Weight (kg), mean (SD)	66.1 (11.9)
Height (cm), mean (SD)	157.0 (6.1)
Body mass index (kg/m^2^), mean (SD)	26.84 (4.8)
Normal weight, N (%)	158 (40.7)
Overweight, N (%)	143 (36.9)
Obesity, N (%)	87 (22.4)
Waist circumference (cm), mean (SD)	85.0 (11.7)
Waist ≥ 88 cm, N (%)	147 (37.9)
Hip circumference (cm), mean (SD)	101.55 (10.6)
Fat mass (kg), mean (SD)	22.43 (7.8)
Percentage fat mass (%), mean (SD)	33.11 (6.6)
Lean mass (kg), mean (SD)	43.70 (5.6)
Percentage lean mass (%), mean (SD)	66.89 (6.6)
Muscle mass (Kg), mean (SD)	41.47 (5.3)
Percentage muscle mass (%), mean (SD)	63.48 (6.2)

^1^ Because of some missing data, not all numbers add up to the total.

**Table 2:** Association between the adherence to the three *a priori* dietary patterns and percentage fat mass, percentage muscle mass, body mass index, and waist circumference in the 362 EPIC-Florence participants, with follow-up dietary information.

	Low Adherence I Tertile	Medium Adherence II Tertile	Good Adherence III Tertile	*p* Trend	Standardized Score Adherence (Continuous) ^c^
**Italian Mediterranean Diet**					
Adherence score range (n. subj.)	0–2 (65)	3–4 (151)	5–9 (146)		
**% fat mass (mean, SD)**	35.7 (5.8)	33.2 (6.4)	31.8 (6.8)		
β (95%CI) ^a^	1	**−2.65 (−4.51; −0.79)**	**−3.62 (−5.48; −1.76)**	**<0.0001**	**−1.25 (−1.90; −0.60)**
β (95%CI) ^b^	1	**−2.47 (−4.32; −0.63)**	**−3.41 (−5.25; −1.57)**	**0.001**	**−1.13 (−1.77; −0.48)**
**% muscle mass (mean, SD)**	61.0 (5.5)	63.4 (6.0)	64.7 (6.5)		
β (95%CI) ^a^	1	**2.51 (0.74; 4.3.)**	**3.42 (1.66; 5.19)**	**<0.0001**	**1.18 (0.56; 1.80)**
β (95%CI) ^b^	1	**2.34 (0.59; 4.10)**	**3.23 (1.48; 4.97)**	**0.001**	**1.07 (0.45; 1.68)**
**Body mass index (mean, SD)**	28.3 (4.3)	26.9 (4.7)	26.0 (4.8)		
β (95%CI) ^a^	1	**−1.38 (−2.72; −0.03)**	**−2.19 (−3.53; −0.85)**	**0.002**	**−0.88 (−1.34; −0.41)**
β (95%CI) ^b^	1	−1.19 (−2.50; 0.12)	**−1.97 (−3.27; −0.66)**	**0.003**	**−0.76 (−1.22; −0.31)**
**Waist Circumference (mean, SD)**	89.0 (10.9)	85.1 (11.7)	83.0 (11.5)		
β (95%CI) ^a^	1	**−3.83 (−7.13; −0.53)**	**−5.31 (−8.60; −2.02)**	**0.003**	**−2.13 (−3.28; −0.98)**
β (95%CI) ^b^	1	**−3.44 (−6.70; −0.19)**	**−4.86 (−8.09; −1.62)**	**0.005**	**−1.89 (−3.02; −0.76)**
**Greek Modified Mediterranean Diet**					
Adherence score range (n. subj.)	0–3 (112)	4–5 (147)	6–8 (103)		
**% fat mass (mean, SD)**	33.9 (6.1)	33.5 (6.2)	31.7 (7.4)		
β (95%CI) ^a^	1	0.15 (−1.75; 1.45)	**−1.89 (−3.63; −0.14)**	**0.04**	**−0.70 (−1.37; −0.04)**
β (95%CI) ^b^	1	0.10 (−1.48; 1.68)	−1.25 (−3.00; 0.51)	0.17	−0.47 (−1.14; 0.20)
**% muscle mass (mean, SD)**	62.8 (5.8)	63.1 (5.9)	64.8 (7.0)		
β (95%CI) ^a^	1	0.13 (−1.38; 1.65)	**1.77 (0.12; 3.43)**	**0.04**	**0.66 (0.03; 1.30)**
β (95%CI) ^b^	1	−0.11 (−1.61; 1.39)	1.17 (−0.50; 2.83)	0.18	0.44 (−0.20; 1.07)
**Body mass index (mean, SD)**	27.9 (4.6)	26.7 (4.4)	25.9 (5.2)		
β (95%CI) ^a^	1	−0.90 (−2.04; 0.24)	**−1.66 (−2.91; −0.41)**	**0.01**	**−0.59 (−1.06; −0.11)**
β (95%CI) ^b^	1	−0.66 (−1.77; 0.46)	−1.10 (−2.33; 0.14)	0.08	−0.39 (−0.87; 0.08)
**Waist Circumference (mean, SD)**	87.1 (11.8)	84.9 (11.0)	82.9 (12.1)		
β (95%CI) ^a^	1	−1.53 (−4.34; 1.28)	**−3.48 (−6.56; −0.41)**	**0.03**	−1.18 (−2.35; 0.002)
β (95%CI) ^b^	1	−1.01 (−3.78; 1.75)	−2.26 (−5.34; 0.81)	0.15	−0.74 (−1.92; 0.43)
**Dietary Approaches to Stop Hypertension (DASH)**					
Adherence score range (n. subj.)	14–23 (107)	24–27 (126)	28–37 (129)		
**% fat mass (mean, SD)**	33.8 (6.2)	33.1 (6.4)	32.5 (7.1)		
β (95%CI) ^a^	1	−0.65 (−2.33; 1.03)	−1.04 (−2.74; 0.67)	0.24	−0.60 (−1.28; 0.08)
β (95%CI) ^b^	1	−0.18 (−1.86; 1.49)	−0.41 (−2.12; 1.30)	0.63	−0.39 (−1.07; 0.29)
**% muscle mass (mean, SD)**	62.9 (5.8)	63.4 (6.1)	64.1 (6.7)		
β (95%CI) ^a^	1	0.61 (−0.99; 2.20)	0.98 (−0.64; 2.59)	0.24	0.57 (−0.08; 1.21)
β (95%CI) ^b^	1	0.17 (−1.42; 1.76)	0.38 (−1.24; 2.01)	0.64	0.36 (−0.28; 1.01)
**Body mass index (mean, SD)**	27.4 (4.4)	27.0 (4.9)	26.2 (4.8)		
β (95%CI) ^a^	1	−0.41 (−1.62; 0.79)	−1.06 (−2.28; 0.16)	0.09	−0.39 (−0.88; 0.09)
β (95%CI) ^b^	1	0.04 (−1.14; 1.22)	−0.48 (−1.69; 0.72)	0.41	−0.18 (−0.66; 0.30)
**Waist Circumference (mean, SD)**	87.4 (12.0)	84.7 (11.9)	83.4 (10.8)		
β (95%CI) ^a^	1	−2.48 (−5.42; 0.46)	**−3.53 (−6.51; −0.55)**	**0.02**	**−1.53 (−2.54; −0.17)**
β (95%CI) ^b^	1	−1.60 (−4.52; 1.33)	−2.36 (−5.34; 0.62)	0.12	−0.93 (−2.11; 0.25)

^a^ Regression model adjusted for age (years, continuous), height (cm), smoking habits (dummy variables for current and former smokers, never-smokers as reference category), number of children (categorical), educational level (dummy variables for high school and university, none/primary school as reference category) and time from menopause (years, continuous). ^b^ Regression model further adjusted for sitting time (categorical variable for ≤2 h/day, >2–≤4 h/day, >4 h/day), recreational activity (MET-hours/week, continuous) and household activity (MET-hours/week, continuous). ^c^ The regression coefficient (β) represents the adjusted difference in body composition measures between two consecutive standardized values of adherence to the three patterns.

**Table 3:** Association between physical activity and percentage fat mass, percentage muscle mass, body mass index, and waist circumference in the 388 EPIC-Florence participants, with follow-up information.

	I Tertile	II Tertile	III Tertile	*p* Trend	PA (Continuous) ^c^
**RECREATIONAL PA ^a^**					
MET-hour/week (n. subj.)	0–13.5 (129)	15.0–27.8 (118)	28.5–171.0 (140)		
**% fat mass (mean, SD)**	34.5 (6.5)	33.3 (6.2)	31.5 (6.7)		
β (95%CI) ^d^	1	−1.27 (−2.86; 0.32)	**−2.06 (−3.64; −0.49)**	**0.01**	**−1.09 (−1.76; −0.42)**
β (95%CI) ^e^	1	−0.79 (−2.44; 0.86)	−1.59 (−3.22; 0.05)	0.06	**−0.89 (−1.62; −0.15)**
**% muscle mass (mean, SD)**	62.1 (6.2)	63.3 (5.9)	65.0 (6.4)		
β (95%CI) ^d^	1	1.20 (−0.30; 2.71)	**1.96 (0.46; 3.45)**	**0.01**	**1.04 (0.40; 1.67)**
β (95%CI) ^e^	1	0.75 (−0.81; 2.32)	1.51 (−0.04; 3.06)	0.06	**0.84 (0.15; 1.54)**
**Body mass index (mean, SD)**	28.3 (5.5)	27.2 (4.6)	25.2 (3.8)		
β (95%CI) ^d^	1	−1.01 (−2.14; 0.12)	**−2.29 (−3.41; −1.17)**	**<0.0001**	**−0.92 (−1.40; −0.43)**
β (95%CI) ^e^	1	−0.76 (−1.92; 0.40)	**−2.11 (−3.26; −0.97)**	**<0.0001**	**−0.91 (−1.43; −0.39)**
**Waist circumference (mean, SD)**	88.2 (11.7)	85.8 (11.9)	81.3 (10.4)		
β (95%CI) ^d^	1	−2.04 (−4.81; 0.73)	**−4.63 (−7.38; −1.88)**	**0.001**	**−1.95 (−3.13; −0.77)**
β (95%CI) ^e^	1	−1.56 (−4.44; 1.31)	**−4.24 (−7.09; −1.39)**	**0.003**	**−1.86 (−3.15; −0.58)**
**HOUSEHOLD PA ^b^**					
MET-hour/week (n. subj.)	0–32.5 (128)	32.9–69.5 (130)	72.7–170.7 (129)		
**% fat mass (mean, SD)**	33.2 (6.9)	32.2 (6.3)	33.8 (6.5)		
β (95%CI) ^d^	1	−0.94 (−2.49; 0.61)	0.04 (−1.56; 1.64)	0.98	−0.09 (−0.74; 0.55)
β (95%CI) ^e^	1	−0.91 (−2.51; 0.70)	0.02 (−1.60; 1.64)	1.00	−0.11 (−0.77; 0.54)
**% muscle mass (mean, SD)**	63.4 (6.5)	64.3 (6.0)	62.8 (6.2)		
β (95%CI) ^d^	1	0.87 (−0.60; 2.35)	−0.04 (−1.56; 1.48)	0.98	0.09 (−0.52; 0.70)
β (95%CI) ^e^	1	0.84 (−0.68; 2.37)	−0.02 (−1.56; 1.52)	1.00	0.11 (−0.52; 0.74)
**Body mass index (mean, SD)**	26.7 (5.2)	26.6 (4.9)	27.2 (4.4)		
β (95%CI) ^d^	1	−0.22 (−1.32; 0.89)	0.10 (−1.04; 1.24)	0.87	0.11 (−0.36; 0.57)
β (95%CI) ^e^	1	−0.18 (−1.31; 0.95)	0.17 (−0.97; 1.31)	0.75	0.17 (−0.30; 0.64)
**Waist circumference (mean, SD)**	85.0 (12.2)	84.2 (12.2)	85.7 (10.6)		
β (95%CI) ^d^	1	−0.84 (−3.55; 1.87)	−0.22 (−2.57; 3.01)	0.90	0.06 (−1.07; 1.20)
β (95%CI) ^e^	1	−0.59 (−3.40; 2.21)	0.30 (−2.53; 3.13)	0.83	0.14 (−1.01, 1.30)
**SITTING TIME**					
Hour/day (n. subj.)	≤2 (170)	>2; ≤4 (162)	>4 (55)		
**% fat mass (mean, SD)**	31.6 (6.7)	33.8 (6.3)	35.9 (5.9)		
β (95%CI) ^d^	1	**1.47 (0.04; 2.90)**	**3.29 (1.31; 5.27)**	**0.001**	**1.18 (0.41; 1.95)**
β (95%CI) ^e^	1	**1.52 (0.04; 3.00)**	**2.77 (0.71; 4.82)**	**0.005**	**0.99 (0.18; 1.81)**
**% muscle mass (mean, SD)**	64.9 (6.4)	62.9 (6.0)	60.9 (5.6)		
β (95%CI) ^d^	1	**−1.40 (−2.76; −0.04)**	**−3.11 (−4.99; −1.23)**	**0.001**	**−1.11 (−1.85; −0.38)**
β (95%CI) ^e^	1	**−1.45 (−2.85; −0.04)**	**−2.62 (−4.57; −0.67)**	**0.005**	**−0.94 (−1.71; −0.17)**
**Body mass index (mean, SD)**	25.6 (4.1)	27.2 (4.7)	29.5 (5.8)		
β (95%CI) ^d^	1	**1.16 (0.14; 2.17)**	**3.00 (1.60; 4.41)**	**<0.0001**	**1.04 (0.48; 1.60)**
β (95%CI) ^e^	1	**1.09 (0.05; 2.13)**	**2.30 (0.85; 3.74)**	**0.001**	**0.79 (0.21; 1.37)**
**Waist circumference (mean, SD)**	82.5 (11.0)	85.6 (11.8)	91.1 (11.4)		
β (95%CI) ^d^	1	1.88 (−0.62; 4.37)	**6.96 (3.51; 10.42)**	**<0.001**	**2.33 (0.97; 3.68)**
β (95%CI) ^e^	1	1.56 (−1.02; 4.14)	**5.41 (1.83; 8.99)**	**0.005**	**1.72 (0.30; 3.15)**

^a^ MET-hours/week of walking, cycling, and fitness activities. ^b^ MET-hours/week of do-it-yourself, housework, gardening activities, and stair climbing. ^c^ Standardized MET-hours/week for recreational activity and household activity. Hours/day for sitting time. ^d^ Regression model adjusted for age (years, continuous), height (cm, continuous), smoking habits (dummy variables for current and former smokers, never-smokers as reference category), number of children (categorical), educational level (dummy variables for high school and university, none/primary school as reference category), time from menopause (years, continuous). ^e^ Regression model further adjusted for total IMD score. Analysis on 362 FEDRA study participants with diet information.

## Data Availability

The datasets generated during and/or analysed during the current study are not publicly available due to participants privacy protection but are available from the corresponding author on reasonable request.

## Abbreviations

The following abbreviations are used in this manuscript:

RDA	Recommended Daily Allowance
PA	Physical Activity
EPIC	European Prospective Investigation into Cancer and Nutrition
BMI	Body Mass Index
FM	Fat Mass
MM	Muscle Mass
WC	Waist Circumference
LSQ	Lifestyle Questionnaire
FFQ	Food Frequency Questionnaire
FEDRA	Florence-EPIC Digital mammographic breast density and breast Risk Assessment
MET	Metabolic Equivalent
HRT	Hormone Replacement Therapy
MDS	Mediterranean Diet Score
GMMD	Greek Modified Mediterranean Diet
IMI	Italian Mediterranean Index
IMD	Italian Mediterranean Diet
DASH	Dietary Approaches to Stop Hypertension
FU	Follow-up
SD	Standard Deviation

## References

[1] WHO-Report of WHO Consultation. https://apps.who.int/iris/handle/10665/42330.

[2] Kuller L.H., Simkin-Silverman L.R., Wing R.R., Meilahn E.N., Ives D.G. (2001). Women’s Healthy Lifestyle Project: A randomized clinical trial: Results at 54 months. Circulation.

[3] Khandelwal S. (2020). Obesity in midlife: Lifestyle and dietary strategies. Climacteric.

[4] Borkan G.A., Hults D.E., Gerzof S.G., Robbins A.H., Silbert C.K. (1983). Age changes in body composition revealed by computed tomography. J. Gerontol..

[5] Lovejoy J.C., Champagne C.M., de Jonge L., Xie H., Smith S.R. (2008). Increased visceral fat and decreased energy expenditure during the menopausal transition. Int. J. Obes..

[6] Flegal K.M., Carroll M.D., Ogden C.L., Curtin L.R. (2010). Prevalence and trends in obesity among US adults, 1999–2008. JAMA.

[7] Kanaley J.A., Sames C., Swisher L., Swick A.G., Ploutz-Snyder L.L., Steppan C.M., Sagendorf K.S., Feiglin D., Jaynes E.B., Meyer R.A. (2001). Abdominal fat distribution in pre- and postmenopausal women: The impact of physical activity, age, and menopausal status. Metabolism.

[8] Neeland I.J., Ross R., Després J.P., Matsuzawa Y., Yamashita S., Shai I., Seidell J., Magni P., Santos R.D., Arsenault B. (2019). Visceral and ectopic fat, atherosclerosis, and cardiometabolic disease: A position statement. Lancet Diabetes Endocrinol..

[9] Silva T.R., Oppermann K., Reis F.M., Spritzer P.M. (2021). Nutrition in Menopausal Women: A Narrative Review. Nutrients.

[10] Romaguera D., Norat T., Mouw T., May A.M., Bamia C., Slimani N., Travier N., Besson H., Luan J., Wareham N. (2009). Adherence to the Mediterranean diet is associated with lower abdominal adiposity in European men and women. J. Nutr..

[11] Gao H.L., Gao H.X., Sun F.M., Zhang L. (2016). Effects of walking on body composition in perimenopausal and postmenopausal women: A systematic review and meta-analysis. Menopause.

[12] Martinez J.A., Wertheim B.C., Thomson C.A., Bea J.W., Wallace R., Allison M., Snetselaar L., Chen Z., Nassir R., Thompson P.A. (2017). Physical Activity Modifies the Association between Dietary Protein and Lean Mass of Postmenopausal Women. J. Acad. Nutr. Diet.

[13] Palli D., Berrino F., Vineis P., Tumino R., Panico S., Masala G., Saieva C., Salvini S., Ceroti M., Pala V. (2003). A molecular epidemiology project on diet and cancer: The EPIC-Italy Prospective Study. Design and baseline characteristics of participants. Tumori J..

[14] Pala V., Sieri S., Palli D., Salvini S., Berrino F., Bellegotti M., Frasca G., Tumino R., Sacerdote C., Fiorini L. (2003). Diet in the Italian EPIC cohorts: Presentation of data and methodological issues. Tumori J..

[15] Istituto Europeo di Oncologia (2017) Banca Dati di Composizione degli Alimenti per Studi Epidemiologici in Italia (BDA). http://www.bda-ieo.it/.

[16] Pols M.A., Peeters P.H., Ocké M.C., Slimani N., Bueno-de-Mesquita H.B., Collette H.J. (1997). Estimation of reproducibility and relative validity of the questions included in the EPIC Physical Activity Questionnaire. Int. J. Epidemiol..

[17] Brage S., Westgate K., Franks P.W., Gradmark A., Tormo Diaz M.J., The InterAct Consortium (2012). Validity of a short questionnaire to assess physical activity in 10 European countries. Eur. J. Epidemiol..

[18] Ainsworth B.E., Haskell W.L., Whitt M.C., Irwin M.L., Swartz A.M., Strath S.J., O Brien W.L., Bassett D.R., Schmitz K.H., Emplaincourt P.O. (2000). Compendium of physical activities: An update of activity codes and MET intensities. Med. Sci. Sports Exerc..

[19] Verney J., Schwartz C., Amiche S., Pereira B., Thivel D. (2015). Comparisons of a Multi-Frequency Bioelectrical Impedance Analysis to the Dual-Energy X-Ray Absorptiometry Scan in Healthy Young Adults Depending on their Physical Activity Level. J. Hum. Kinet..

[20] Trichopoulou A., Orfanos P., Norat T., Bueno-de-Mesquita B., Ocké M.C., Peeters P.H., van der Schouw Y.T., Boeing H., Hoffmann K., Boffetta P. (2005). Modified Mediterranean diet and survival: EPIC-elderly prospective cohort study. BMJ.

[21] Agnoli C., Krogh V., Grioni S., Sieri S., Palli D., Masala G., Sacerdote C., Vineis P., Tumino R., Frasca G. (2011). A priori-defined dietary patterns are associated with reduced risk of stroke in a large Italian cohort. J. Nutr..

[22] Appel L.J., Moore T.J., Obarzanek E., Vollmer W.M., Svetkey L.P., Sacks F.M., Bray G.A., Vogt T.M., Cutler J.A., Windhauser M.M. (1997). A clinical trial of the effects of dietary patterns on blood pressure. DASH Collaborative Research Group. N. Engl. J. Med..

[23] Fung T.T., Chiuve S.E., McCullough M.L., Rexrode K.M., Logroscino G., Hu F.B. (2008). Adherence to a DASH-style diet and risk of coronary heart disease and stroke in women. Arch. Intern. Med..

[24] Galmes-Panades A.M., Varela-Mato V., Konieczna J., Wärnberg J., Martínez-González M.Á., Salas-Salvadó J., Corella D., Schröder H., Vioque J., Alonso-Gómez Á.M. (2019). Isotemporal substitution of inactive time with physical activity and time in bed: Cross-sectional associations with cardiometabolic health in the PREDIMED-Plus study. Int. J. Behav. Nutr. Phys. Act..

[25] Kyle U.G., Gremion G., Genton L., Slosman D.O., Golay A., Pichard C. (2001). Physical activity and fat-free and fat mass by bioelectrical impedance in 3853 adults. Med. Sci. Sports Exerc..

[26] Rosique-Esteban N., Babio N., Díaz-López A., Romaguera D., Alfredo Martínez J., Sanchez V.M., Schröder H., Estruch R., Vidal J., Buil-Cosiales P. (2019). Leisure-time physical activity at moderate and high intensity is associated with parameters of body composition, muscle strength and sarcopenia in aged adults with obesity and metabolic syndrome from the PREDIMED-Plus study. Clin Nutr..

[27] Panagiotakos D.B., Pitsavos C., Stefanadis C. (2006). Dietary patterns: A Mediterranean diet score and its relation to clinical and biological markers of cardiovascular disease risk. Nutr. Metab. Cardiovasc. Dis..

[28] Papavagelis C., Avgeraki E., Augoulea A., Stamatelopoulos K., Lambrinoudaki I., Yannakoulia M. (2018). Dietary patterns, Mediterranean diet and obesity in postmenopausal women. Maturitas.

[29] Cespedes Feliciano E.M., Tinker L., Manson J.E., Allison M., Rohan T., Zaslavsky O., Waring M.E., Asao K., Garcia L., Rosal M. (2016). Change in Dietary Patterns and Change in Waist Circumference and DXA Trunk Fat Among Postmenopausal Women. Obesity.

[30] Pisani P., Faggiano F., Krogh V., Palli D., Vineis P., Berrino F. (1997). Relative validity and reproducibility of a food-frequency dietary questionnaire for use in the Italian EPIC centres. Int. J. Epidemiol..

[31] Boneva-Asiova Z., Boyanov M.A. (2008). Body composition analysis by leg-to-leg bioelectrical impedance and dual-energy X-ray absorptiometry in non-obese and obese individuals. Diabetes Obes. Metab..

[32] Wang J.G., Zhang Y., Chen H.E., Li Y., Cheng X.G., Xu L., Guo Z., Zhao X.S., Sato T., Cao Q.Y. (2013). Comparison of two bioelectrical impedance analysis devices with dual energy X-ray absorptiometry and magnetic resonance imaging in the estimation of body composition. J. Strength Cond. Res..

